# Are we ignoring a black elephant in the Anthropocene? Climate change and global pandemic as the crisis in health and equality

**DOI:** 10.1007/s11625-020-00879-7

**Published:** 2020-11-07

**Authors:** Shinichiro Asayama, Seita Emori, Masahiro Sugiyama, Fumiko Kasuga, Chiho Watanabe

**Affiliations:** 1 National Institute for Environmental Studies, Tsukuba, Japan; 2 Institute for Future Initiatives, University of Tokyo, Tokyo, Japan; 3 Future Earth Secretariat, Tokyo, Japan

**Keywords:** Climate change, COVID-19, Pandemic, Anthropocene, Health and equality, Empathy

## Abstract

Climate change and coronavirus pandemic are the twin crises in the Anthropocene, the era in which unsustainable growth of human activities has led to a significant change in the global environment. The two crises have also exposed a chronic social illness of our time—a deep, widespread inequality in society. Whilst the circumstances are unfortunate, the pandemic can provide an opportunity for sustainability scientists to focus more on human society and its inequalities, rather than a sole focus on the natural environment. It opens the way for a new normative commitment of science in a time of crises. We suggest three agendas for future climate and sustainability research after the pandemic: (1) focus on health and well-being, (2) moral engagement through empathy, and (3) science of loss for managing grief.

## Introduction: the two crises in an emergency room

We cannot solve a crisis without treating it as a crisis.—Greta Thunberg

In December 2018, a young Swedish girl, Greta Thunberg, made a public speech at the Twenty-Fourth Conference of Parties to the United Nations Framework Convention on Climate Change in Katowice, Poland. After that, she soon became a global icon of new-generation climate activism. The above catchphrase alluded to the disjuncture between a growing sense of urgency of global climate risk and a sluggish pace of policy actions on reducing greenhouse gas emissions. It effectively captures the public’s frustration at inadequate governmental responses to the climate crisis. But this now famous line of hers also raises an important question: What kind of crisis climate change is and what does “treating a crisis as a crisis” really mean?

In many senses, the coronavirus disease 2019 (COVID-19) pandemic testifies to what policy responses to a crisis look like. In times of crisis, decisive intervention is required to prevent catastrophic damage from unfolding (Hulme et al. 2020; Lidskog et al. 2020). The mathematical models predicted that unmitigated virus outbreak would lead to a precipitous rise in death rates from COVID-19 (Adam 2020). The distressing model predictions forced many governments to swiftly impose emergency measures that were unimaginable in normal times, such as shutting down schools and business, prohibiting public gatherings and closing national borders. Just like rescuing a patient taken to a hospital emergency room, these draconian measures were justified simply because it is ethically unacceptable not to save lives from *preventable* deaths (Orr and Wolff 2015). Zinn (2020) called COVID-19 a “monstrous threat” that “legitimises significant restrictions to people’s freedom and is justified by the ethics to keep everyone safe”.

However, the COVID-19 pandemic has also revealed an underlying chronic social illness of the world: pervasive social and economic inequalities (Zinn 2020). A blanket implementation of population-wide preventive health interventions such as national lockdowns hit the already vulnerable groups of people hardest, thereby exacerbating the inequality in health (Frohlich and Potvin 2008; Lancet 2020; Newland 2020). Not only are they in a high risk of contracting the virus due to a lack of space for physical distancing but also they bear the secondary cost of deprived livelihood by lockdowns. The pandemic put vulnerable populations in double jeopardy.

This inequality paradox involved with COVID-19 response shows clearly the *social* nature of the crises we face (Hulme et al. 2020). Insomuch as governments need to control the virus and hence maintain the healthcare system for patient treatment, they must also protect the economy to help those who are affected by secondary risks of the pandemic not to fall into impoverishment (McKee and Stuckler 2020). At the centre of responding to the coronavirus crisis are health and equality (or lack thereof), both of which are inextricably tied to each other.

In this essay, we argue that both climate change and global pandemic are the result of the unsustainable growth of human activities. The two crises are a true testament to the Anthropocene, a new geological epoch of our own making (Crutzen 2002). The pandemic can provide an unfortunate yet rare opportunity to take more seriously the concept of health in environmental sustainability research. A focus on health and well-being in the sustainability challenges helps us to pay judicious attention to human society and inequalities within it rather than the natural environment. The fundamental ethical challenge is, however, to create a shared sense of responsibility for protecting the health of “unknown others” through empathy.

## A new reality of human life in the Anthropocene

When the novel coronavirus emerged first in Wuhan, China, at the end of 2019, causing severe respiratory diseases, it was a big surprise. In the eyes of many people, COVID-19 appeared to be a sudden crisis came out from nowhere. However, disease ecologists have been warning, for a long time, of the growing risk of emerging infectious diseases—in particular, zoonotic viruses that spill over from non-human animals into humans (Daszak et al. 2000; Jones et al. 2008). Anthropogenic land-use change (i.e. deforestation caused by agricultural land acquisition) has led to wildlife habitat loss, which has subsequently increased the risk of zoonoses spillover (Allen et al. 2017; Gibb et al. 2020). So, in the eyes of disease ecologists, even though how exactly the virus jumped from animals to humans is yet unknown, the emergence of novel coronavirus (originating from bats) was a predictable, imminent threat. Just like climate scientists have anticipated that climate change would become a grave threat to humanity.

A New York Times opinion columnist, Thomas Friedman, described the COVID-19 pandemic as a “black elephant”: a cross between a “black swan” (an unlikely, unexpected event with enormous ramifications) and the “elephant in the room” (a looming disaster that is visible to everyone, yet no one wants to address).^1^ This character of “black elephant” events resonates with what risk analysists called the risk of *Cassandra*—a Greek mythological figure who was given the gift of prophecy but her true prophecies would not be believed (Renn and Klink 2004). Climate change and biodiversity loss are examples of such highly probable, catastrophic risks that no one is willing to acknowledge because of a slow, prolonged effect of environmental degradation.

Despite a striking difference of temporality (Lidskog et al. 2020), climate change and global pandemic are the twin crises in the Anthropocene. The common, underlying cause of both is a planetary-scale, anthropogenic change in the natural environment—the Earth’s atmosphere and terrestrial land, respectively. An exponential growth in scale and speed of human activities (“Great Acceleration”) constitutes the two crises as truly global challenges of the Anthropocene (Steffen et al. 2015). They are equally “black elephants” in that scientists have long been sounding the alarm about a calamity caused by human actions but the governments paid woefully insufficient attention, despite knowing that ignorance will lead to dire consequences.

Importantly, what makes COVID-19 a pandemic is hyper-globalisation of human travel (Zhu et al. 2020). For example, if this coronavirus emerged in human society, say, in rural China of 1980s, it may still cause serious disease outbreak locally but will not probably become a *pandemic* as it is today. It is our highly-mobile modern lifestyle—globalised travel and rapid urbanisation—that provides warp speed for the virus spreading around the world. Ironically, as Zinn (2020) said, COVID-19 “developed into a major social threat not mainly due to its ability to kill but by challenging our way of living”. Asymptomatic or presymptomatic transmission (He et al. 2020) allows the virus to spread without detection—an epidemiologist called it the “invisible pandemic” (Giesecke 2020). This points to that disease eradication (i.e. global reduction of infection to zero cases) is awfully difficult and that without vaccines, the world will be under constant threat of virus resurgence (Heywood and Macintyre 2020). The punctuated normalcy that was imposed as a *temporary* state of emergency is then morphed into a “new normal” under the pandemic.

The pandemic’s nature of global systemic risk (Renn et al. 2019) suggests that even the countries that succeeded in controlling or eliminating the virus such as New Zealand are not free from interference. To prevent future outbreaks, they need to maintain a strict border-control policy (Baker et al. 2020), which costs their economy immensely. As the Director-General of the World Health Organization (WHO), Tedros Adhanom Ghebreyesus, remarked at the press conference on COVID-19 response, the reality of the pandemic is that “no one is safe until everyone is safe”.^2^ The same is true for climate change. The groundswell of climate change and its policy response will swallow up everyone. In other words, this inescapability of global interconnectedness is our new ontology of being humans in the Anthropocene (Lövbrand et al. 2015).

## Proclaiming the Anthropocene: planetary boundaries, planetary health

While climate change and global pandemic can be equally understood as great challenges in the Anthropocene, their manifestation has been pronounced differently due to a difference in disciplinary concerns between environmental sustainability research and public health research.

In the field of global environmental change research, climate change is often used as a shorthand for global sustainability challenges. The major concern of researchers in this field is largely concentrated upon regulating the stability of the Earth System within “planetary boundaries”—a safe operating space for humanity (Rockström et al. 2009; see also Steffen et al. 2020). The concept of planetary boundaries has become a dominant framework for integrating the physical science of understanding the processes of the Earth System into the social science of studying the policy and governance response at the global level. It also provides a powerful narrative that captures the growing worries among scientists (and the public at large) over the alarming rate of environmental change which might be reaching beyond the Earth’s capacity to absorb human impacts and sustain life within it.

Some such scientists now characterise the 2 °C of warming of global temperature as a planetary threshold that, if crossed, will push the Earth System onto an irreversible pathway towards “Hothouse Earth” (Steffen et al. 2018). A call for declaring a climate emergency (Lenton et al. 2019) is, in this respect, a *political* attempt by scientists to warn about how little time is left to avoid such a catastrophic scenario. A problem is however that there is little attention paid to the human societies, which are seen as an integral part of the Earth System (Steffen et al. 2020). Like a well-known German sociologist Ulrich Beck (2010) said: “If ‘the environment’ only includes everything which is not human, not social, then the concept is sociologically empty.”

Richard Horton, the editor-in-chief of *The Lancet*, the prestigious medical journal, noticed this problem with a technical approach to the Anthropocene challenge:

The planetary boundaries approach is powerful. But it repeats the mistake of saying that it is something outside of us that should be the object of our concern—carbon dioxide, acidified oceans, and so on. Not so. The object of our concern should be us. The seeds of our vulnerability lie within ourselves, not the disturbed natural systems around us. (Horton 2015).

While inspired by the planetary boundaries concept, Horton and other public health professionals recognise the important role of health at the centre of the sustainability challenges. They see human and environmental health as two sides of one coin. This has culminated in the emergence of the concept of “planetary health”, which is defined as “the health of human civilisation and the state of the natural systems on which it depends” (Whitmee et al. 2015; see also Myers 2017).

Notably, the planetary health approach shifts a focus onto the quality of human life and the socio-political institutions that shape human responses to planetary threats (Horton 2013). A manifesto issued by Horton and his colleagues summarises the underlying motives behind this new concept:

Planetary health is an attitude towards life and a philosophy for living. It emphasises people, not diseases, and equity, not the creation of unjust societies. We seek to minimise differences in health according to wealth, education, gender, and place. (Horton et al. 2014).

In a sense, this is a plea for seeing the climate and sustainability challenges through a lens of health. The health concept has been used in several different ways in the context of ecology and the environment (Mallee 2017). There are mounting concerns over the impacts of climate change on human health (Watts et al. 2019). Nonetheless, health seems to remain not a primary concern among sustainability researchers.

And then, the world was struck by the COVID-19 pandemic. Now health is the basic question on everyone’s mind. This may be a renewed opportunity to take more seriously the health perspective in sustainability research. The concept of planetary health can provide a common language for transdisciplinary approaches to bridging a gap between environmental sustainability and public health.^3^ Health can be used as an eclectic concept that combines the diverse ways of knowing from different strands of disciplines and concerns.

## Social and economic inequality at the heart of the crisis

According to the WHO definition, health is not merely the absence of disease but “a state of complete physical, mental and social well-being” (WHO 1946). During the pandemic, the people’s health is affected not only by the virus itself but also by the measures for controlling the virus. Therefore, a pandemic response must look beyond the virus and carefully look at the social and economic dimensions of illness. It is required to address both the clinical care of symptoms and the societal roots of illness (Mendenhall 2017).

Crucially, the effects of the pandemic depend more on the pre-existing social conditions of countries than on the biological nature of the virus. The coronavirus pandemic turned into a serious economic crisis because the political economy *before* the pandemic (e.g. a decade-long austerity, a growth in insecure job employment) allowed the virus to deprive economically precarious people of their livelihoods (McKee and Stuckler 2020). One of the most striking paradoxes of the COVID-19 response is that those who work at low-skilled jobs were suddenly seen as “essential workers” during the lockdown. While these “essential workers” needed to keep going out to maintain the functioning of social systems, the wealthier individuals who are usually most mobile in normal times became least mobile during the lockdown because they could afford to self-quarantine at home (Weill et al. 2020; see also Bonaccorsi et al. 2020). Put simply, social distancing is the privilege of the rich while the poor suffer from it.

The virus has thus revealed the egregious socio-economic inequalities within and between countries (Zinn 2020). There is already abundant evidence that the coronavirus disproportionately affected the most socially marginalised groups—racial minorities (Yancy 2020), temporary migrant workers (Koh 2020), sex workers (Platt et al. 2020), prisoners or persons in custody (Kinner et al. 2020), homeless people (Tsai & Wilson 2020), and so on and so forth. Domestic violence against women has soared around the world since lockdowns began (Wenham et al. 2020). In developing countries, lockdowns would do more harm than good to the people in poverty, especially children (Broadbent et al. 2020).

The immediate, medical losses directly associated with COVID-19 are countable, if not completely, as shown by the fatalities statistics. However, the long-term consequences of structural social inequalities worsened by the pandemic are difficult to measure numerically, and hence often tend to “kill silently” (Zinn 2020). An early policy response to the pandemic that focused upon “flattening the curve” of viral spread, informed primarily by mathematical models, failed to take into account the wider social and economic costs of lockdowns (Caduff 2020), which are necessarily conditioned by pervasive inequalities.

This policy failure to address social inequalities might partly explain a recent global surge in anti-racism protests by the *Black Lives Matter* (BLM) movement in the wake of the killing of George Floyld, an African American male who also lost his job due to stay-at-home orders in Minnesota, the US. The protest for racial justice under the pandemic somewhat resonates with the *Fridays For Future* (FFF) movement, the global youth strike for climate justice, inspired by Greta Thunberg. As an outrage at unjust treatment by police against Black Americans brought many people into the streets, a sense of injustice to future generations mobilised young people to walk out of school to fight for the climate. Both anti-racism and climate protests represent “moral outrage” against social injustice—an *empathetic* (not personal) anger about the unfairness and injustice (Antadze 2020). They convey a moral emotion about the unfair treatment of the other.

The two protests of BLM and FFF movements teach us how badly the politics failed to address socio-economic inequality in responding to the crises. These protesters are angry at the institutional disregard for the suffering of the most vulnerable in the society. They can be understood as an outcry against the “politics of apathy” (Antadze 2018). The US President Donald Trump is the most outrageous example of such political apathy. But scientists are not free from any shadow of guilt. While scientists are instrumental to enacting a policy for reducing greenhouse gas emissions or controlling the virus spread, they might turn a blind eye to the existing social inequalities and the societal losses that are difficult to show in numbers. This is the fundamental ethical challenge that science is facing in the midst of *social* crises.

Vaccines would probably end the COVID-19 pandemic at some point in the future but we do not have a vaccine for inequality. We also have to be vigilant about the possibility that the availability of a vaccine will cause new geopolitical tensions (Fidler 2020), which might end up in widening the divide between haves and have-nots. In addition to vaccine development, a decisive action on reducing inequality is required. If borrowed Thunberg’s phrase, we cannot solve the inequality without treating it as a *crisis*.

## The way forward: a need for empathy and science of loss

A crisis of any kind tells eloquently who we are or what kinds of society we live in. A crisis is a “mirror into which we can look and see exposed both our individual selves and our collective societies” (Hulme 2009). Both climate change and the coronavirus pandemic attest to the fact that we are now living in the Anthropocene, the era in which unsustainable growth of human activities has caused a significant change in the global environment. But at the same time, the two crises have exposed a deep, widespread inequality in the twenty-first-century society—a chronic social illness of our time. As inequality in society is intrinsically coupled with changes in the ecological condition (Hamann et al. 2018), addressing socio-economic inequality is imperative for global sustainability science. In light of this, we suggest the three research agendas for future climate and sustainability research after the pandemic.

First, focus on health and well-being of the human systems—social, political and economic—and their interaction with the Earth’s natural systems. As argued above, the concept of planetary health, moving away from an obsession with planetary boundaries and climate tipping points, will provide a useful point of reference for interdisciplinary and transdisciplinary research that can cut across traditional boundaries between environmental sustainability and public health. The health perspective is, for example, instrumental in better integrating the risk of emerging infectious diseases within sustainable development planning (Di Marco et al. 2020). Strengthening health systems through universal health coverage is essential for several UN Sustainable Development Goals (SDGs), such as no poverty, quality education and gender equality (Kieny et al. 2017). Since health and equality are closely linked to each other, protecting human and environmental health is conducive to the central pledge of SDGs: “leave no one behind”.

Second, sustainability research needs to have a moral engagement with the “unknown other” through empathy (Antadze 2019). As Antadze (2019) noted, a new reality in the age of the Anthropocene requires us to rethink the nature of *togetherness* as an openness to the other who is unknown but may suffer the unfair consequences of our own actions. The totality of climate change and global pandemic means that we have to take responsibility for the suffering of the other and develop an imaginative capacity through empathy to emotionally engage with the other. Empathy is sharing of another’s emotional state or putting oneself in someone’s shoes. However, sustainability science so far appears to turn away from empathising with the people’s sufferings in real life. Now is a time of reckoning. Scientists should hold up mirrors which reveal the contradictions and limitations of and within science, including systemic racism in science (Nature 2020; Thorp 2020). By doing so, sustainability research should have a new normative commitment to building collective empathy for the vulnerable other.

Third, related to the second point, sustainability research needs a science of loss for managing grief (Barnett et al. 2016). An uncomfortable, unsettling truth of climate change and global pandemic is that the loss of people, places, practices, and so many others is somewhat unavoidable. Whilst the policy to prevent loss is no doubt needed, embracing—rather than denying—the possibility of loss may perhaps help people better overcome or come to terms with grief and loss. A science of loss is to understand what kind of loss would be intolerable to people, how a loss might arise through changes in the natural environment and, when the loss is unavoidable, help people to manage grief and remember loss (Barnett et al. 2016). Importantly, as Barnett et al. (2016) said, many phenomena that people value are often *incommensurable* with no possible substitutes; hence, there can be no effective compensation for their loss. As the New York Times wrote in an obituary of a hundred thousand lives lost to coronavirus, it is an “incalculable loss”.^4^ To condole with the people in grief can be the role of science. This means that the research has to go further into an *emotional* engagement with stakeholders, more than just that for producing knowledge, at which is often aimed in transdisciplinary sustainability research (Lang et al. 2012).

Taken together, a crisis necessitates a transformation of the science toward more socially and ethically engaged research. The coronavirus crisis has put a normal life of people on hold, requiring to not go back to the old normal but move onto the new normal. With a sense of urgency of the climate crisis, many people understand the politics must go beyond business as usual. Likewise, this is—and should be—a moment of change in our way of doing the science in times of crisis. It will be a moral failure of the science to keep ignoring the “black elephant” in an emergency room.

## Footnotes

1 Thomas Friedman, “We Need Herd Immunity From Trump and the Coronavirus”, The New York Times, 25 April 2020. https://www.nytimes.com/2020/04/25/opinion/coronavirus-immunity-trump.html

2 WHO, “WHO Director-General's opening remarks at the media briefing on COVID-19—18 August 2020”. https://www.who.int/dg/speeches/detail/who-director-general-s-opening-remarks-at-the-media-briefing-on-covid-19---18-august-2020

3 On the other hand, epidemiologists and disease ecologists call for the “one health” approach to an integrative understanding of human-animal-environmental dynamics for the prevention of zoonotic diseases (Bonilla-Aldana et al. 2020; Zhu et al. 2020).

4 The New York Times, “Remembering the 100,000 Lives Lost to Coronavirus in America”, 24 May 2020. https://www.nytimes.com/interactive/2020/05/24/us/us-coronavirus-deaths-100000.html
